# Investigations on Leucas cephalotes (Roth.) Spreng. for inhibition of LPS-induced pro-inflammatory mediators in murine macrophages and in rat model

**DOI:** 10.17179/excli2014-667

**Published:** 2015-04-10

**Authors:** Neeraj K. Patel, Mohd. Shahid Khan, Kamlesh K. Bhutani

**Affiliations:** 1 Department of Natural Products, National Institute of Pharmaceutical Education and Research (NIPER), Sector 67, S.A.S Nagar, Mohali-160062, Punjab (INDIA)

**Keywords:** Leucas cephalotes, GC-MS, anti-inflammatory, nitric oxide, tumor necrosis factor-alpha, interleukin-1beta

## Abstract

Silica gel column chromatography fractionation of the dichloromethane extract (LCD) of *Leucas cephalotes *(Roth.) Spreng. led to the isolation of five compounds namely β-sitosterol (**1**) + stigmasterol (**2**), lupeol (**3**), oleanolic acid (**4**) and laballenic acid (**5**). Also, gas chromatography-mass spectrometry (GC-MS) analysis of sub-fraction (LCD-F1) of this extract showed the presence of eleven (**6**-**16**) compounds. In addition to this, **3**-**5** and LCD-F1 were evaluated for lipopolysachharide (LPS)-induced nitric oxide (NO), tumor necrosis factor (TNF)-α and interleukin (IL)-1β production in RAW 264.7 and J774A.1 cells. Results directed that **4** and **5** were found to inhibit these mediators at half maximal inhibitory concentration of 17.12 to 57.20 μM while IC_50_ for LCD-F1 was found to be 15.56 to 31.71 μg/mL. Furthermore, LCD at a dose of 50, 100 and 400 mg/Kg was found to reduce significantly LPS induced tumor necrosis factor (TNF)-α and interleukin (IL)-1β production in female Sprague Dawley (SD) rats. All the results findings evoked that the anti-inflammatory effects of *Leucas cephalotes* is partially mediated through the suppression of pro-inflammatory mediators and hence can be utilized for the development of anti-inflammatory candidates.

## Introduction

*Leucas cephalotes* (Roth.) Spreng. (Family: Lamiaceae) also known as 'Dronapushpi' in Sanskrit, is an edible rainy season weed. In Ayurveda, it has been recommended for inflammation, psoriasis, scabies, chronic skin eruptions, edema, diaphoresis, chronic malaria, asthma, eye diseases, jaundice, paralysis and obstinate urinary troubles (Dash, 1987[8]; Bhoria and Kainsa, 2013[6]; Nadkarni, 2007[16]; Chandel et al., 1996[7], Miyaichi et al., 2006[15]). The plant is reported to have anti-filarial activity, anti-bacterial, anti-diabetic activity and hepato-protective activity (Bhoria and Kainsa, 2013[6]; Rastogi and Mehrotra, 1991[21]; Bavarva and Narasimhacharya, 2010[3]). Leaves of this plant is applied in the form of paste for snake bite treatment and also recommended in case of chronic rheumatism (Samy et al., 2008[22]; Bhoria and Kainsa, 2013[6]). Yadav et al., (2012[23]) reported that non-polar fraction of methanolic extract of *L. cephalotes *significantly reduced the increased levels of TNF-α, IL-1β and IL-6 in mouse ear tissue homogenate. Baburao et al. (2010[1]) also found that at doses of 200 and 400 mg/Kg body weight *p. o* methanolic extract of this plant demonstrated anti-inflammatory activity in a carrageenan induced rat paw edema model. Considering the anti-inflammatory potential of *Leucas cephalotes* and its related species *viz.* *Leucas aspera* (Kripa et al., 2011[14]; Baburao et al., 2010[1]), we evaluated previously the dichloromethane (LCD), ethyl acetate (LCE) and methanol extracts (LCM) of this plant for *in vitro* pro-inflammatory cytokines (TNF-α and IL-1β) and nitric oxide (NO) inhibition in RAW 264.7 cells. Results revealed that LCD exhibited significant inhibition of NO, TNF-α and IL-1β production with an IC_50 _of 49.3, 46.8 and 49.8 µg/mL respectively as compared to LCE and LCM (IC_50 _= 50 to > 100 µg/mL) (Patel et al., 2014[19]). In the light of this, the present study was designed with an aim to isolate and identify compounds from LCD and afterwards evaluate them towards inhibition of pro-inflammatory mediators.

## Materials and Methods

### Plant material

*Leucas cephalotes *(Roth.) Spreng. (whole plant) was procured from Arya Vastu Bhandar, Dehradun, Uttarakhand, India. A voucher no; NIP-NPM-CD-017 has been deposited at the Department of Natural Products, NIPER, Punjab, India after proper authentication by a botanist. 

### Instruments

Ultraviolet (UV) spectra were measured on a DU^®^ 7400 spectrophotometer (Beckman, München, Germany). ^1^H and ^13^C Nuclear magnetic resonance (NMR) spectra were recorded on Ultrashield 400 MHz and 100 MHz spectrometer respectively (Bruker DPX, Faellanden, Germany). Mass spectra were recorded on a mass spectrometer (Thermo Quest Finnigan, San Jose, CA, USA). For *in vitro* experiments, ultracentrifuge (Sigma, St. Louis, MO, USA), CO_2_ incubator (WTC Binder, Tuttlingen, Germany), Biosafety cabinet (Clean air, Chennai, India), autopipettes, ELISA plate reader (Labsystems, Helsinki, Finland) and Neubauer chamber (HBG, Gießen, Germany) were used. Extracts were dried *in vacuo* using a vacuum rotary evaporator (Buchi R-210, Flawil, Switzerland). 

### Chemicals and reagents

Silica gel (230-400 mesh; Merck (Mumbai, India) was used for column chromatography. TLC plates, pre-coated with silica gel 60 F_254_ thickness 0.2 mm and solvents (laboratory grade) were obtained from Merck (Darmstadt, Germany). Dulbecco's modified Eagle's medium (DMEM), fetal bovine serum (FBS), phosphate buffered saline (PBS), 3-(4,5-dimethyl-2-thiazolyl)-2,5-diphenyl-2*H*-tetrazolium bromide (MTT), antibiotic solution and other chemicals for the biological experiments were purchased from Hi-media Limited (Mumbai, India). Lipopolysaccharide (*Escherichia coli* 026:B6) (LPS), dimethyl sulfoxide (DMSO), dexamethasone and curcumin were purchased from Sigma Chemical Co. (St. Louis, MO, USA). Mouse and rat TNF-α and IL-1β ELISA kits were supplied by Krishgen Biosystems (Mumbai, India). 

### Extraction and isolation 

Dried and pulverized whole plants (2.8 Kg, 20-30 mesh) was subjected to sequential maceration with 6 L (3x) hexanes (Hex), dichloromethane (DCM), ethyl acetate (EtOAc) and methanol (MeOH) for 72 h at 25 °C to yield hexanes (LCH, 23.6 g), dichloromethane (LCD, 32.3 g), ethyl acetate (LCE, 21.6 g) and methanol (LCM, 18.3 g) extracts respectively. Vacuum liquid chromatography (VLC) of LCD (48.7 g) over silica gel (230-400 mesh) column chromatography (CC) was performed using a stepwise gradient of hexanes containing increasing amounts of EtOAc (from 0 % up to 100 %). Three pooled fractions; LCD-F1 (22.67 g, 0-25 % EtOAc/Hex), LCD-F2 (1.24 g, 25-40 % EtOAc/Hex) and LCD-F3 (8.37 g, 40-60 % EtOAc/Hex) were obtained on the basis of similar thin layer chromatography (TLC) profiles. LCD-F1 was found to be oily in nature and hence subjected for GC-MS analysis after derivatization. LCD-F2 (1.24 g) was fractionated by CC on silica gel (230-400 mesh) using a gradient of hexanes and ethyl acetate (4:1-7:3) and on crystallized with aqueous methanol provided **1 **and** 2 **(76 mg, mixture). Similarly, LCD-F3 (8.37 g) was loaded on a repeated silica gel column (230-400 mesh) and eluted gradient wise, using hexanes and EtOAc (13:7-1:1) yielded **3 **(18 mg),** 4 **(38 mg) and** 5** (10 mg). 

### GC-MS analysis

GC-MS Clarus 600 Gas chromatograph with Clarus 600 C mass spectrometer, column: ELITE 5MS 0.25 id, 30 m (Perkin Elmer) was used for analysis. Inlet line temperature was 270 °C and source temperature was 200 °C. Injector temperature: 220 °C, Runtime: 36.50 min, gas chromatography started at 35 °C saturated it for 10 min and then gradual increase of temperature by 10 °C/min up to 200 °C and then here saturated for 10 min and the obtained chromatogram was scanned by mass spectrometry by mass range scanned 10-600 EI. 10 mg of LCD-F1 was dissolved in 20 mL dry methanol, then 5 to 6 drops of concentrated sulphuric acid (H_2_SO_4)_ was added, subsequently refluxed at 50 °C to 80 °C for 12 h. Thereafter, methanol was evaporated and residue was neutralized by adding sodium bicarbonate solution, and then extracted with chloroform. Organic layer was taken, dried with sodium sulphate and then concentrated under reduced pressure using rotary evaporator.

### Cell culture, cell viability and nitric oxide release assay

RAW 264.7 and J774A.1 cells were obtained from the National Centre for Cell Sciences (NCCS, Pune, India) and were cultured in 250 mL culture flasks containing DMEM supplemented with heat inactivated 10 % FBS, 10,000 units/mL pencillin and 10 ng/mL streptomycin in 0.9 % saline, in a CO_2_ incubator (5 % CO_2_ in air) at 37 °C. MTT assay was carried out to check the cell viability by measuring the purple formazan formation in the mitochondria of living cells. NO release in the culture medium was quantified using the Griess reaction as previously reported by us (Bhandari et al., 2014[4][5]; Patel and Bhutani, 2014[18]). Briefly, cells were pre-incubated with different concentrations of the test samples for 1 h at 37 °C, and then stimulated with 1 µg/mL of LPS. After 24 h incubation, supernatants were mixed with an equal amount of modified Griess's reagent. After further incubation for 30 min, optical density (OD) was measured at 540 nm. 

### TNF-α and IL-1β assays

Cytokine levels in supernatants were measured by sandwich immunoassays using the supplier's protocol (Krishgen Biosystems, Mumbai, India) (Patel and Bhutani, 2014[17]; Patel et al., 2014[19]; Bhandari et al., 2014[4][5]). Briefly, cells were pre-incubated with different concentrations of the test samples for 1 h at 37 °C, and then stimulated with 1 µg/mL of LPS (*Escherichia coli *026:B6) in a 96 well microtitre plate. In the case of TNF-α and IL-1β, the cells were further incubated for 6 h and 12 h, respectively and cytokine level in supernatants were measured at 450 nm.

### Measurement of TNF-α and IL-1β production in plasma of SD rats

The experimental study was approved by the Institute Animal Ethics Committee (IAEC) under approval number IAEC/13/46 and performed according to the guidelines of the Committee for the Purpose of Control and Supervision of Experiments on Animals (CPCSEA) given on animal experimentation. Female Sprague Dawley (SD) rats (aged 6-8 weeks, 200-250 g, *n = *5 in each group) were issued from the Central Animal Facility (CAF), NIPER and separated into three different groups (control, test and a positive control group) and were permitted to have free water and normal diet *ad libitum*. Cytokines (TNF-α and IL-1β) concentrations were determined as per the method previously described by us (Patel and Bhutani, 2014[17][18]). Briefly, a control group received *p. o.* vehicle (10 % Tween 80), test group received LCD (50, 100 and 400 mg/kg) and a positive control group received dexamethasone (5 mg/kg) respectively at an interval of 12 h. LPS (5 mg/Kg) was administered *i. p.* after 2 h of the final dose. After LPS administration, blood samples were collected later 2 h and 6 h in case of TNF-α and IL-1β respectively. Animals were monitored for any unusual behavior and signs of toxicity at treated doses of test samples. Sandwich ELISA kits specific for rats were used to determine the cytokines (TNF-α and IL-1β) levels in the plasma.

### Statistical analysis

The results are expressed as the mean ± SD for all triplicates experiments. Half maximal concentration was calculated from concentration *vs* percent inhibition curves. Statistical analysis was performed by One-Way ANOVA followed by a Dunnett's post test (commercially available software SigmaStat 3.5). A level of *p* < 0.05 was used as the criterion of statistical significance.

## Results and Discussion

### Characterization of isolated compounds

**Figure 1:**
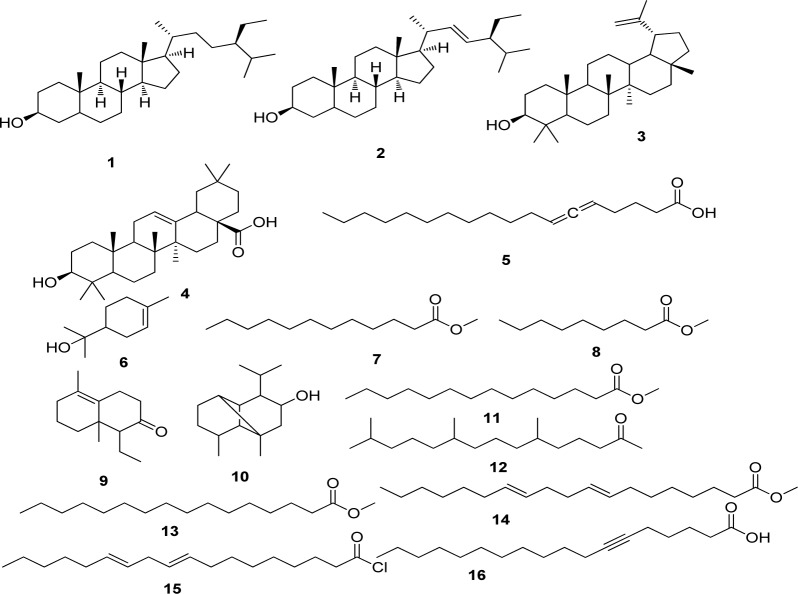
Compounds isolated (1-5) from DCM extract of *Leucas cephalotes* and identified (6-16) from LCD-F1 by GC-MS

**Figure 2:**
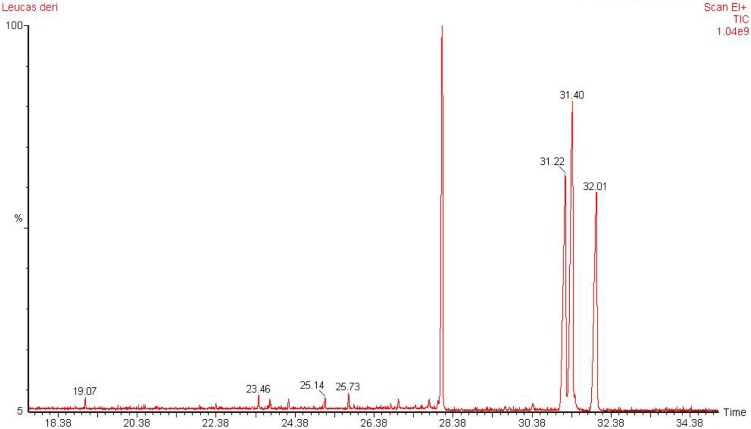
GC-MS profile for LCD-F1 of *Leucas cephalotes*

The present study deals with the identification of the anti-inflammatory constituents present in *L. cephalotes* using inhibition of LPS induced NO, TNF-α and IL-1β levels in RAW 264.7 and J774A.1 cells. LCD exhibited significant inhibition of NO, TNF-α and IL-1β production with an IC_50 _of 49.3, 46.8 and 49.8 µg/mL respectively as compared to LCE and LCM (IC_50 _= 50 to > 100 µg/mL) (Patel et al., 2014[19]). Five compounds namely β-sitosterol (**1**) + stigmasterol (**2**) (mixture) (Patel et al., 2014[19]), lupeol (**3**), oleanolic acid (**4**) (Bhandari et al., 2014[4][5]; Galipalli et al., 2014[10]) and laballenic acid (**5**) (Bagby et al., 1965[2]) were isolated and identified from LCD (Figure 1(Fig. 1)). Also, GC-MS analysis of sub-fraction (LCD-F1) revealed the presence of eleven (**6**-**16**) compounds (Table 1(Tab. 1), Figures 1(Fig. 1) and 2(Fig. 2)).

### Effects of fraction/isolated compounds on NO production and cell viability in macrophages

LCD-F1 and isolated compounds from *L. cephalotes* were evaluated for the inhibition of LPS induced NO production using RAW 264.7 and J774A.1 cells. As visualized from the Table 2(Tab. 2), among the tested compounds, oleanolic acid (**4**) (IC_50 _= 20.74 to 25.22 µM) and laballenic acid (**5**) (IC_50 _= 22.03 to 23.21 µM) were found to be slightly more potent than lupeol (**3**) (IC_50_=34.51 to 37.36 µM) in inhibiting NO production in macrophages. Similarly, LCD-F1 possessed the minimal inhibitory concentration of 23.45 to 25.57 μg/mL for inhibition of NO production. We have previously tested β-sitosterol (**1**) + stigmasterol (**2**) (mixture) for inhibition of pro-inflammatory markers in macrophages (Patel et al., 2014[19]) and hence not included here. All the tested samples owned appreciable cell viability of > 84.23 % at concentration of 100 μM/100 μg/mL.

### Effects of fraction/isolated compounds on pro-inflammatory cytokines production in macrophages

Table 3(Tab. 3) presents the TNF-α and IL-1β inhibitory effects of LCD-F1 and isolated compounds from *L. cephalotes* on RAW 264.7 and J774A.1 cells. LCD-F1 inhibited TNF-α and IL-1β with an IC_50_ ranging from 15.56 to 19.46 μg/mL and 28.51 to 31.71 μg/ mL respectively. Lupeol (**3**) (IC_50 _= 12.33 to 14.28 µM), oleanolic acid (**4**) (IC_50 _= 17.12 to 19.22 µM) and laballenic acid (**5**) (IC_50 _= 17.21 to 18.32 µM) were found to be active against TNF-α production in macrophages. In case of IL-1β inhibition, tested compounds were found to be lesser active with half maximal concentration of 32.71 to 89.21 µM as compared to TNF-α inhibition. There are reports for anti-inflammatory properties of sesquiterpenes, fatty acids and their derivatives (Keyzers and Davies-Coleman, 2005[13]; El-Demerdash, 2011[9]; Held et al., 2007[12]; Hamdan et al., 2013[11]) and hence, these components present in LCD-F1 are responsible for the inhibition of pro-inflammatory mediators (NO, TNF-α and IL-1β).

### Inhibitory effects of LCD on pro-inflammatory cytokines in SD female rats

**Figure 3:**
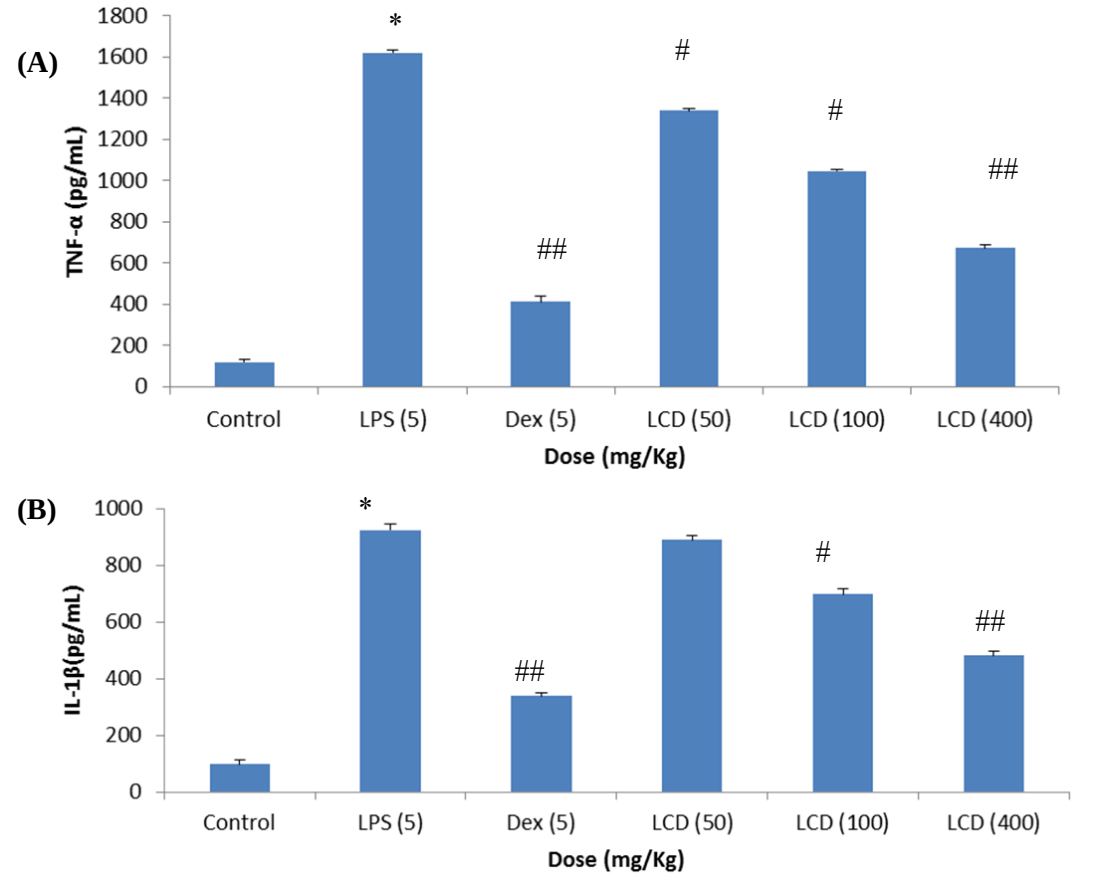
Effect of LCD on plasma levels of (A) TNF-α and (B) IL-1β in SD rats {* (*p* < 0.001) = *vs* control; #(*p* < 0.05), ##(*p* < 0.001) = *vs* LPS}. Values are expressed as mean ± SD; *n*= 5 animals. The data was analyzed by One way of ANOVA test followed by Dunnett's test. LPS: lipopolysaccharide, LCD: dichloromethane extract of *L. cephalotes *whole plant

LCD was further evaluated in female SD rats for *in vivo* inhibition of pro-inflammatory cytokines. Animals were observed for any altered reaction after the subjection of LPS or/and test samples. It was found that test samples and LPS do not cause any toxic effects. As shown in Figure 3(Fig. 3) (A and B) there is a substantial increase (*p* < 0.001) in the TNF-α (1623 pg/mL) and IL-1β (912 pg/ mL) levels in the LPS induced groups with respect to the normal group (< 87 pg/mL). Dexamethasone (standard drug) at a dose of 5 mg/Kg was found to suppress significantly (*p* < 0.001) TNF-α production by 75.8 % (394 pg/mL) and IL-1β production by 63.0 % (338 pg/mL) respectively. LCD at treated dose of 50, 100 and 400 mg/Kg manifested significant (*p* < 0.05 and 0.001) reduction of TNF-α levels by 1340, 1045, and 673 pg/mL and IL-1β levels by 889, 697 and 481 pg/mL respectively. Our results corroborated with the study conducted by Yadav et al., (2012[23]) where non-polar fraction of methanolic extract of *L. cephalotes *significantly reduced the increased levels of TNF-α, IL-1β and IL-6 in mouse ear tissue homogenate. Baburao et al. (2010[1]) also reported that at doses of 200 and 400 mg/Kg body weight *p. o* methanolic extract of this plant demonstrated an anti-inflammatory activity in a carrageenan induced rat paw edema model. Hence, it can be estimated that mostly the non-polar components *viz*. fatty acids, sesquiterpenes, steroids and triterpenes present in LCD are responsible for the anti-inflammatory effects of *L. cephalotes.*

To summarize the results, anti-inflammatory effect of *L. cephalotes* is partially mediated through the suppression of pro-inflammatory mediators and hence can be utilized in part or along with available anti-inflammatory drugs for the treatment of inflammatory disorders.

## Conflict of interest

The authors declare that there is no conflict of interest.

## Acknowledgements

NKP worked as Department of Science and Technology (DST)-Inspire Senior Research Fellow for the doctoral program. Authors are grateful to the Director, NIPER, S.A.S. Nagar, India for providing the required research facilities for the present work. 

## Figures and Tables

**Table 1:**
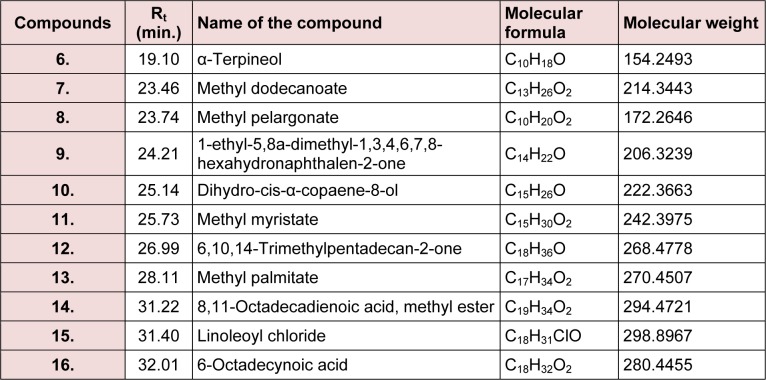
Compounds identified in LCD-F1 of *Leucas cephalotes* by GC-MS

**Table 2:**
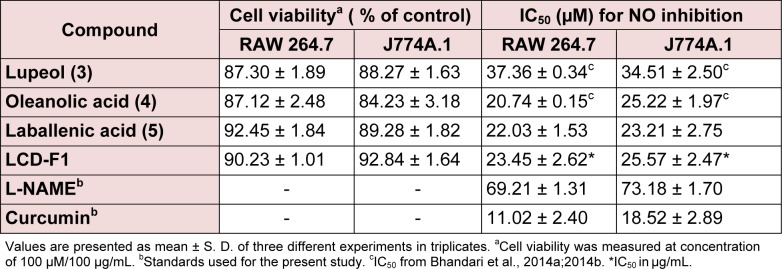
Cell viability and NO inhibitory effects of isolated compounds/fraction from *L. cephalotes *

**Table 3:**
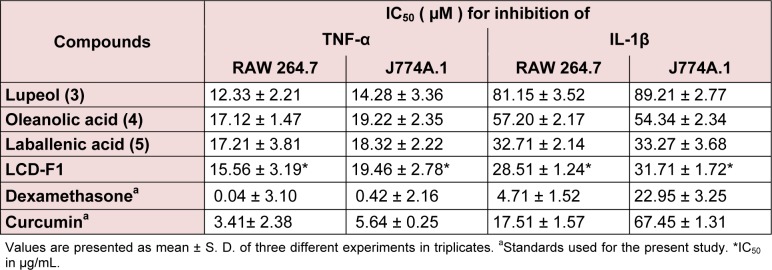
TNF-α and IL-1β inhibitory effects of isolated compounds/fraction from *L. cephalotes *
